# Nitric Oxide Releasing Nanoparticles Are Therapeutic for *Staphylococcus aureus* Abscesses in a Murine Model of Infection

**DOI:** 10.1371/journal.pone.0007804

**Published:** 2009-11-12

**Authors:** George Han, Luis R. Martinez, Mircea Radu Mihu, Adam J. Friedman, Joel M. Friedman, Joshua D. Nosanchuk

**Affiliations:** 1 Department of Physiology and Biophysics, Albert Einstein College of Medicine, Bronx, New York, United States of America; 2 Department of Medicine, Division of Infectious Diseases, Albert Einstein College of Medicine, Bronx, New York, United States of America; 3 Department of Microbiology and Immunology, Albert Einstein College of Medicine, Bronx, New York, United States of America; 4 Department of Medicine, Division of Dermatology, Montefiore Medical Center, Bronx, New York, United States of America; David Geffen School of Medicine at University of California Los Angeles, United States of America

## Abstract

*Staphylococcus aureus* (*SA*) is a leading cause of a diverse spectrum of bacterial diseases, including abscesses. Nitric oxide (NO) is a critical component of the natural host defense against pathogens such as *SA*, but its therapeutic applications have been limited by a lack of effective delivery options. We tested the efficacy of a NO-releasing nanoparticle system (NO-np) in methicillin-resistant *SA* (MR*SA*) abscesses in mice. The results show that the NO-np exert antimicrobial activity against MR*SA* in vitro and in abscesses. Topical or intradermal NO-np treatment of abscesses reduces the involved area and bacterial load while improving skin architecture. Notably, we evaluated pro- and anti-inflammatory cytokines that are involved in immunomodulation and wound healing, revealing that NO-np lead to a reduction in angiogenesis preventing bacterial dissemination from abscesses. These data suggest that NO-np may be useful therapeutics for microbial abscesses.

## Introduction

*Staphylococcus aureus* (*SA*) is an immotile Gram positive coccus that frequently colonizes human nasal membranes and skin. This bacterium is responsible for the majority of superficial and invasive skin infections, resulting in over 12,000,000 outpatient/emergency room visits [1] and 400,000 hospital admissions annually in the USA [2]. Notably, in a study performed across multiple emergency departments across the USA, MRSA was isolated from 61% of abscesses and 53% of purulent wounds [3]. Furthermore, certain *SA* clinical strains have recently evolved resistance to vancomycin, an antibiotic to which staphylococci have previously been uniformly sensitive. Although the vancomycin-resistant strains remain rare, methicillin-resistant strains (MR*SA*) are increasingly common [4], highlighting the urgent need to develop new approaches for the treatment of *SA* infections. Furthermore, the incidence of community acquired strains of MRSA has markedly increased over the past several years [5].

Topically applied nitric oxide (NO) is a potentially useful preventive and therapeutic strategy against superficial skin infections, including MR*SA* infections [6], [7]. In the healthy state and under pathologic conditions, it is well established that NO maintains skin homeostasis by regulating circulation, ultraviolet-mediated melanogenesis, sunburn erythema, and the maintenance of the protective barrier against microorganisms [8]. Notably, NO modulates immune responses [9] and is a significant regulator of wound healing [10]. We have recently developed an inexpensive and stable NO releasing platform using nanotechnology based on a silane hydrogel [7]. Moreover, our platform benefits from the presence of chitosan, which also has antimicrobial activity [11], [12]. Chitosan is a polymer derived from crustacean exoskeletons that binds to and disrupts the cell wall and membrane of microorganisms due to its cationic charge in weakly acidic environments.

In this study, we investigate the applicability of topically applied NO via nanoparticles (NO-np) to MR*SA* subcutaneous abscesses. Based on our previous work [7], [13] and the fact that NO can penetrate skin, we hypothesized that NO-np can be microbicidal to bacteria in an *in vivo* setting. To validate this hypothesis, we investigated the biological impact of NO-np on MR*SA* using a mouse infection model.

## Results

### NO-np Inhibits MR*SA* Growth

**Figure 1:**
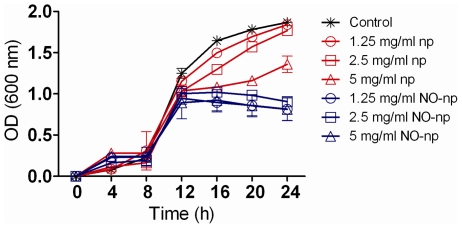
NO-np inhibits MR*SA* growth *in vitro*. The effect of NO in MR*SA* growth kinetics after 24 h co-culture was determined using Bioscreen C analysis. MR*SA* was grown in the absence or presence of nanoparticles with NO (NO-np) or without NO (np). Each point represents the average of four measurements and error bars denote standard deviations.

The effect of NO-np on MR*SA* growth was determined in real-time for 24 h using Bioscreen C analysis (Fig. 1). NO-np significantly reduced bacterial growth after 16 h co-incubation when compared with MR*SA* grown with 1.25 (NO released from nps corresponding to 18.75 nM for the initial peak; 12.5 nM for steady state) and 2.5 (37.5 nM for the initial peak; 25 nM for steady state) mg/mL of np or medium alone. Interestingly, MR*SA* grown with 5 (75 nM for the initial peak; 50 nM for steady state) mg/mL np resulted in a significant reduction in bacterial growth after 16 h, which is likely due to the effects of chitosan on the np. However, after this time point the bacteria subjected to 5 mg/mL np increased their replication rate.

### Intradermal and Topical Administrations of NO-np Decreased MR*SA* Burden in Subcutaneous Abscesses

**Figure 2:**
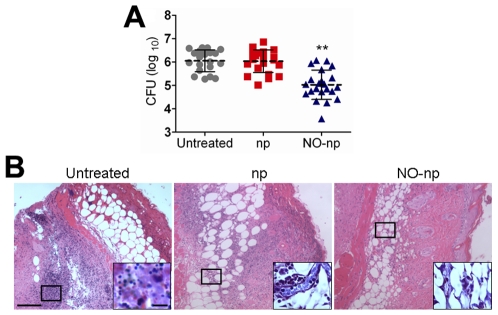
NO-np killed MR*SA* in subcutaneous abscesses. (**A**) Abscess bacterial burden (CFU; colony forming units) in mice infected subcutaneously with 10^7^ MR*SA* and treated with NO-np is significantly lower than untreated or np-treated mice (*n* = 20 abscesses per group). Each point represents an abscess. Dashed lines are the averages of the results and error bars denote standard deviations. Asterisks denote *P* value significance (** *P*<0.001) calculated by analysis of variance and adjusted by use of the Bonferroni correction. (**B**) Histological analysis of Balb/c mice untreated MR*SA*-infected, np-treated MR*SA*-infected, and MR*SA*-infected treated with NO, day 4. Mice were infected with 10^7^ MR*SA*. Representative H&E-stained sections of the skin lesions are shown with the *Insets* showing Gram staining of MR*SA*. Scale bars: 25 µm.

To maximize the exposure of the NO-np to the bacteria in the abscesses, we initially tested the compound by intradermally administering NO-np into the MR*SA* abscesses. MR*SA*-infected NO-np treated abscesses showed significantly lower microbial burden than untreated or np-treated abscesses (*P*<0.001) (Fig. 2A). To determine whether NO-np was similarly therapeutic via topical application, mouse abscesses were topically-treated with NO-np. Similar to the intra-abscess injection of NO-np, topical treatment with NO-np significantly lowered microbial burden (CFU (log_10_) 4.85±0.29) in comparison to untreated (CFU (log_10_) 5.59±0.45) (*P*<0.01) or np-treated abscesses (CFU (log_10_) 5.50±0.24) (*P*<0.001).

Histological examinations of MR*SA*-infected abscesses, both untreated and np-treated, displayed a dense, neutrophil rich infiltrate along with extensive cell necrosis (Fig. 2B). Tissue Gram stains of these samples revealed large numbers of Gram positive cocci (Fig. 2B; insets). Tissue sections from abscesses of NO-np treated MR*SA*-infected mice revealed less suppurative inflammation along with increased fibrin deposition and a lower number of bacteria (Fig. 2B; insets). Consistent with our prior work [13], NO-np or np did not show toxicity on uninfected animals (data not shown).

### NO-np Decreased Subcutaneous Abscess Area in Mice

**Figure 3:**
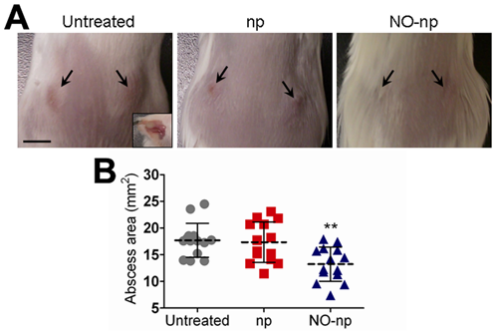
NO-np decreases subcutaneous abscess area in mice. (**A**) Abscesses of Balb/c mice untreated MR*SA*-infected, np-treated MR*SA*-infected, and MR*SA*-infected treated with NO-np, day 4. Arrows denote abscesses. *Inset* shows a representative purulent abscess 4 days after MR*SA* infection. Scale bar: 5 mm. (**B**) Abscess area analysis of Balb/c mice subcutaneous lesions. Abscesses were infected with MR*SA* and untreated or treated in the absence or presence of NO. Each point represents an abscess. Dashed lines are the averages of the results for fourteen measurements, and error bars denote standard deviations. Asterisks denote *P* value significance (** *P*<0.001) calculated by analysis of variance and adjusted by use of the Bonferroni correction.

The effect of NO-np on abscess formation in Balb/c mice was investigated (Fig. 3). Application of NO-np directly into subcutaneous abscesses via injection decreased the area of these structures significantly (Fig. 3A). At day 4, the area of MR*SA*-infected abscesses treated with NO-np reached ∼13.24 mm^2^, whereas the area of abscesses of MR*SA*-infected untreated or treated with nanoparticles only was ∼17.70 mm^2^ (*P*<0.001) and ∼17.34 mm^2^ (*P*<0.001), respectively (Fig. 3B). In separate experiments, topical application of NO-np similarly decreased the size of the MR*SA* abscesses. At day 4, the area of MR*SA*-infected abscesses topically treated with NO-np was ∼17.02 mm^2^ whereas the area of abscesses of MR*SA*-infected untreated or treated with nanoparticles only was ∼20.10 mm^2^ and ∼21.90 mm^2^ (*P*<0.05), respectively. Notably, complete abscess resolution of MR*SA*-infected and NO-np-treated mice is 7±2 days in comparison with ∼14–21 days in the other infected groups (data not shown).

### NO-np Preserves Skin Architecture in Abscesses by Preventing MR*SA* Collagen Degradation

**Figure 4:**
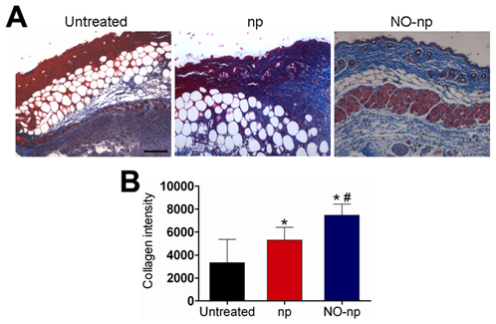
NO-np prevents MR*SA* collagen degradation in subcutaneous abscesses. (**A**) Histological analysis of Balb/c mice untreated MR*SA*-infected, np-treated MR*SA*-infected, and MR*SA*-infected treated with NO-np, day 4. Mice were infected with 10^7^ bacterial cells. The blue stain indicates collagen. Scale bar: 25 µm. (**B**) Quantitative measurement of collagen intensity in 20 representative fields of the same size for untreated MR*SA*-infected, np-treated MR*SA*-infected, and MR*SA*-infected treated with NO-np. Bars are the averages of the results, and error bars denote standard deviations. Asterisks denote *P* value significance (*, *P*<0.05 in comparing MR*SA*-infected np and untreated control groups; **, *P*<0.001 in comparing MR*SA*-infected NO-np and untreated control groups; #, *P*<0.01 in comparing MR*SA*-infected np and NO-np groups) calculated by analysis of variance and adjusted by use of the Bonferroni correction.

The mechanisms through which the NO-nps accelerate wound healing were further explored by examining whether NO-nps prevented collagen degradation by MR*SA* in infected tissue (Fig. 4). Collagen content was highest in infected wounds treated with NO-nps (Fig. 4A). The dispersed blue stain indicated thicker and more mature tissue collagen formation in abscesses treated with NO-np, suggesting that NO-np exposure maintained dermal architecture through bacterial clearance, and ultimately guarding collagen. Figure 4B is a morphometric analysis of the data shown in Figure 4A.

### NO-np Induces Cytokine Expression by the Host

We measured the cytokine response in the abscesses of mice subcutaneously infected with MR*SA*. At day 4 post-infection, abscess tissue of infected mice treated with NO-np contained significantly higher quantities of TNF-α, IFN-γ, IL-12, IL-1β, MCP-1, and TGF-β than that of np or untreated mice (Table 1). Also, abscess tissue of infected mice treated with NO-np exhibited reduced levels of IL-4 and IL-10. Moreover, control and np-treated mice did not show evidence of a consistent pattern of cytokine expression.

**Table 1:** Cytokine levels in abscesses of mice.

	Cytokine levels (pg/mL) (Average ± SEM)							
	IL-12	IFN-γ	TNF-α	IL-4	IL-10	TGF-β	IL-1β	MCP-1
Untr	618.79±3.84	152.5±1.32	85±0.5	56.76±2.32	58.66±7.86	911.76±3.38	45.06±1.56	36.54±0.95
Np	592.57±4.47	158.07±2.15	83.7±0.31	74.17±5^a^	51.87±7.14	977±1.3	52.78±2.12	19.56±0.35^b^
NO-np	812.04±4.34^a^	251.14±1.57^a^	118.83±0.45^a^	51.35±2.6	38.1±8.07^b^	1349±1.51^a^	83.82±1.14^a^	121.39±1.43^a^

*n* = 7 mice per group.

a Value significantly greater than the value for control mice (*P*<0.05).

b Value significantly less than the value for control mice (*P*<0.05).

### NO-np Reduces Angiogenesis in MR*SA* Abscesses

**Figure 5:**
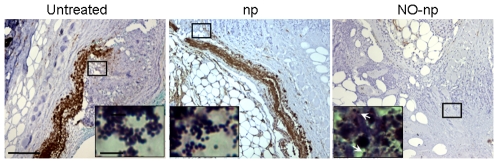
NO-np decreases vascularization in the setting of MR*SA* abscesses. Histological analysis of untreated, np-treated and NO-np treated MR*SA*-infected Balb/c mice, day 4. Mice were infected with 10^7^ MR*SA*. The brown staining indicates vascularization. Representative CD34-immunostained sections of the skin lesions are shown with the *Insets* representing Gram of MR*SA*. White arrows denote Gram positive cocci. Scale bars: 25 µm.

We investigated the effect of NO-np on angiogenesis in MR*SA* abscesses by measuring the expression of CD34 (Fig. 5). Tissue sections from untreated or np-treated MR*SA*-infected murine abscesses displayed dense vascularization in tissue in which high numbers of bacteria were present (Fig. 5; insets). In contrast, abscesses of NO-np treated MR*SA*-infected mice, showed minimal formation of blood vessels in the setting of reduced bacterial burden (Fig. 5; insets).

### NO-np Attenuates Cellular Damage of RHE Tissue Infected with MR*SA*

**Figure 6:**
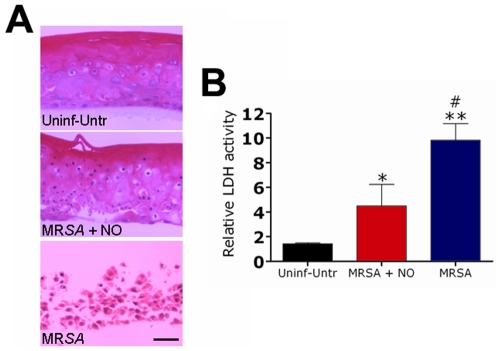
NO reduce cellular damage of reconstituted epidermal tissue infected with MR*SA*. (**A**) NO-np attenuate cellular damage of reconstituted epidermal tissue infected with MR*SA*. Reconstituted human epidermis control group had no bacteria or nanoparticles, reconstituted human epidermis infected with *SA*, and reconstituted human epidermis infected with *SA* and treated with NO-np. Scale bar, 10 µm. (**B**) Relative LDH activity measured in the tissue culture supernatant by reconstituted human epidermal tissue after 24 h co-culture with *SA* in the absence or presence of NO-np. Bars are the averages of the results for three measurements, and error bars denote standard deviations. Asterisks denote *P* value significance (*, *P*<0.05 in comparing the MR*SA*+NO-np group with the control group; **, *P*<0.001 in comparing the MR*SA* group with control group; #, *P*<0.01 in comparing the MR*SA* group with MR*SA*+NO-np group) calculated by analysis of variance and adjusted by use of the Bonferroni correction.

The control reconstituted human epidermal (RHE) tissue was selected as it is representative of a normal keratinizing epithelium, comprised of basal cells, spinous keratinocytes, granular keratinocytes with basophilic keratohyaline granules, and keratinizing cells, yet without the capacity for recruitment of inflammatory cells that may exacerbate host tissue damage (Fig. 6A). RHE infected with MR*SA* and treated with NO-np exhibited less severe histological changes than untreated tissue (Fig. 6A). Infection with MR*SA* caused severe attenuation of the RHE by 24 h (“atrophy”) with flattening of all layers of the epithelium. There were no keratohyaline granules (Fig. 6A). The numbers of apoptotic cells increased and there was separation between the basal cells and the overlying spinous keratinocytes. There was severe intracellular edema, particularly in the basal cells. In general, the basal cells and spinous keratinocytes in the epithelium were disorganized. There was clear cleft formation between the low cuboidal basal cells and the overlying spinous keratinocytes. This suprabasilar separation could be section artifact, but this occurred reproducibly suggesting that this phenomenon was associated with progressive superficial infection and structural disorganization. Additionally, the tissues show signs of bacterial penetration.

To quantify cell injury, we compared the levels of LDH released after MR*SA* was co-cultured with RHE in the presence or absence of NO nanoparticles for 24 h (Fig. 6B). Co-culture in the absence of NO-np caused a significant change in LDH level. The LDH results correlated with the tissue damage seen by light microscopy. MR*SA* co-culture in the absence of NO-np had the highest level of the LDH and tissue damage, whereas the tissue infected with bacteria and treated with NO-np had significantly lower levels of LDH and less histological damage.

## Discussion

The growing danger of life-threatening infections and the rising economic burden of resistant bacteria have created a demand for new antibacterial therapeutics. The use of nanoparticles as delivery vehicles for bactericidal agents represents a new paradigm in the design of antibacterial therapeutics. Nitric oxide (NO), a diatomic free radical that plays a key role in the natural immune system response to infection [14], represents an alternative approach in the design of antibacterial nanoparticles. Moreover, we recently showed the applicability of topically applied NO through nanoparticles (NO-np) to superficial *SA* skin infections [13]. In the present study, NO-np is effective at killing pathogenic MR*SA* in infected subcutaneous abscesses when applied topically or intradermally. The avascular and biofilm-like nature of bacterial abscesses and the colonization/infection of resistant microbial species have undermined the efficacy of conventional antibiotic therapy. Furthermore, the need of surgical drainage of abscesses underscores the need for novel therapeutic approaches, such as the sustained delivery of NO.

In our previous work, we showed that NO-np were effective against 20 different clinical isolates of MRSA (11 strains) and MSSA (9 strains) using CFU analysis [13]. However, to better define the activity of the NO-np on MR*SA*, a real time-killing assay was performed in tryptic soy broth (TSB) using a Bioscreen C device. Such real time-kill studies offer valuable information regarding the temporal efficacy of antimicrobial agents [15]. Conventional antibacterial susceptibility tests such as the minimum bactericidal concentration (MBC) assays do not allow for acute temporal studies. In TSB cultures of MR*SA*, NO-np significantly reduced bacterial growth when compared with MR*SA* grown with np only or medium alone. Interestingly, the highest concentration of np without NO resulted in a significant reduction in bacterial growth at 16 h, which is likely due to the effects of chitosan on the np core. The antimicrobial activity of chitosan, a hydrophilic biopolymer industrially obtained by *N*-deacetylation of crustacean chitin, has been observed against a wide variety of microorganisms including fungi, algae, and bacteria [11].

Our studies demonstrated that NO-np have significant antimicrobial efficacy in the setting of MR*SA* abscesses. Bacterial abscesses can lead to serious complications including sepsis, tissue damage, and death. Abscesses are difficult to treat due to their tendency to prevent immune cells from attacking or reaching the causative microorganism. In this regard, since NO is a gas, it is able to diffuse into the abscess thereby reducing bacterial burden and the area of subcutaneous abscesses by inducing vascular permeability and vasodilation. Furthermore, NO stimulates the infiltration of immune cells such as neutrophils, macrophages, and lymphocytes [16]. NO-np can potentially induce a protective immune response capable of containing the infection, therefore preventing systemic dissemination.

Another benefit of NO-np treatment of MR*SA* abscesses is the stimulation of healing by induction of collagen deposition. NO promotes wound healing through collagen secretion by fibroblasts [17], [18], [19]. Acceleration of wound healing by nitric oxide donors has been demonstrated previously [20]. Indeed, accelerated healing of gastric ulcers has been demonstrated in rats treated with a nitric oxide-releasing derivative of diclofenac, and a similar effect could be observed by treating the rats with glyceryl trinitrate [21]. Further, Thornton *et al*. demonstrated that collagen deposition was enhanced in wounded rats transfected *in vivo* with the gene for inducible nitric oxide synthase, and the increased nitric production within the wound milieu preceded the observed increase in collagen synthesis [22]. Additionally, we recently suggested that topically applied NO-np might also prevent collagen degradation by bacterial collagenases through a reduction in bacterial burden [13]. Bacteria impair repair processes by producing toxic byproducts and competing with cells for oxygen and nutrients [23].

In treating MR*SA* subcutaneous abscesses with NO-np, as proposed in our study, we showed that NO-np can modulate immune responses to facilitate the reduction of bacterial burden. This is the highest priority in treating chronic and non-healing abscesses since a persistent infection and accumulation of bacterial antigens, as commonly seen in microbial abscesses, can impair host responses [24]. A persistent infection can further disrupt the normal process of healing by impairing recruitment and migration of immune cells to the site of injury, leading to abnormal levels of cytokines and growth factors [25]. The success of the chronic abscess elimination process by NO-np stimulation possibly depends on cytokines and growth factors involved in a complex integration of signals that coordinate cellular processes. For instance, the presence of high levels of pro-inflammatory IL-12 might be related to its anti-angiogenic activity, which can block the formation of new blood vessels, therefore, preventing bacterial dissemination to other organs or tissues. This is accomplished by increasing production of IFN-γ, which in turn inhibits endothelial cell motility and vascularization. In this regard, tissue sections stained for CD34, a marker for blood vessel formation, demonstrated that NO-np reduces angiogenesis in MR*SA* abscesses. In fact, the downregulation of IL-10, which can antagonize IFN-γ effects [26], suggest that NO-np might also impede MR*SA* dissemination within phagocytes. Additionally, other pro-inflammatory cytokines/chemokines, particularly TNF-α, IL-1β, and MCP-1 were up-regulated by NO-np. Sustained expression of these pro-inflammatory effector molecules permits a prolonged presence of neutrophils, macrophages, lymphocytes, and mast cells in the chronic abscess contributing to an effective inflammatory response and bacterial clearance [27]. Nitric oxide activates latent TGF-β, which is believed to regulate collagen deposition by fibroblasts [28], [29]. Furthermore, elevated levels of IFN-γ, TNF-α and IL1-β have been shown to directly increase the levels TGF-β [30].

In summary, application of NO as topical agents has been used with success in augmenting wound healing and reducing wound bacterial burden [6], [13]. The presented data show that NO-np have both antimicrobial and wound-healing properties. Overall, the presented results show that the topical or intradermal application of NO-np is highly effective against subcutaneous MR*SA* abscesses in a murine model. Conceivably, this technology might be used as a potential therapy prior to or in addition to surgical drainage of bacterial abscesses. It is further possible that the NO-np could be useful in the treatment of deep abscesses via injection into tissues such as the lung or liver. Interestingly, the NO-np appear to significantly stimulate the immune system. These results suggest that this platform has the potential to serve as a novel, easily administered class of topical antimicrobials for the treatment of subcutaneous infections and abscesses.

## Materials and Methods

### Ethics Statement

All animal studies were conducted according to the experimental practices and standards approved by the Animal Welfare and Research Ethics Committee at the Albert Einstein College of Medicine.

### Methicillin-Resistant *SA* 6498

Methicillin-resistant *SA* (MR*SA*) 6498 is a clinical isolate selected for this study because the strain is resistant to diverse commonly used antibiotics and it was extensively utilized in our prior superficial wound model [13]. The strain was collected from a patient's wounds at Montefiore Medical Center, Bronx, NY and stored in BHI broth (BBL, Cockeysville, MD) containing 40% glycerol at −80°C until use, and then was grown in Tryptic Soy broth (TSB; MP Biomedicals, LLC, Solon, OH) overnight at 37°C with rotary shaking at 150 rpm. Growth was monitored by measuring the optical density at 600 nm (Bio-Tek, Winooski, VT).

### Synthesis of NO-np

The synthesis of NO-np was recently reported along with its potential as a treatment for superficial infections [7], [13]. Briefly, a hydrogel/glass composite was synthesized using a mixture of tetramethylorthosilicate (TMOS), polyethylene glycol (PEG), chitosan, glucose, and sodium nitrite in 0.5 M sodium phosphate buffer (pH 7). The nitrite was reduced to NO within the matrix due to the glass properties of the composite effecting redox reactions initiated with thermally generated electrons from glucose. After redox reaction, the ingredients were combined and dried with a lyophilizer, resulting in a fine powder comprised of nanoparticles containing nitric oxide. Once exposed to an aqueous environment, the hydrogel properties of the composite allow for opening of water channels inside the particles, facilitating the release of the trapped NO over extended time periods. Control nanoparticles (np) consist of the same formulation as NO-np, but without the addition of sodium nitrate.

### Susceptibility of MR*SA* 6498 to NO-np

To evaluate the susceptibility of MR*SA* to NO-np, TSB was inoculated with a fresh colony grown on BHI plates and suspended in 1 mL of medium. A suspension of 100 µL of MR*SA* was transferred to a 200-well plate with 100 µL of TSB per well containing NO-np or np (1.25, 2.5, or 5 mg/mL). Bacteria and nanoparticles were incubated at 37°C for 24 h. Wells containing bacteria with TSB alone was used as a control. Growth was estimated by measuring optical density at 600 nm every 30 min using a Bioscreen C microplate reader (Growth Curves USA, Piscataway, NJ).

### *In Vitro* Infection Model

Reconstituted human tissues were obtained from SkinEthic Laboratory (Nice, France) and reconstituted by incubating in serum-free, MCDB 153 defined medium (Colonetics, San Diego, CA, USA), containing 5 µg/mL insulin, 1.5 mM CaCl_2_, and 0.4 µg/mL hydrocortisone without antibiotics in 6-well tissue culture plates (Corning Inc., Corning, NY, USA) according to the manufacturer's instructions. The reconstituted human epidermis (RHE) was generated from human keratinocytes derived from juvenile foreskin obtained during surgery. Cultures were fully differentiated three-dimensional tissues, grown on the air-liquid interface for 14 days prior to delivery. The tissues were inoculated with 10^7^ MR*SA* 6498 in 100 µL PBS by adding the cell suspension to the 1 mL cell culture medium in the absence or presence of 5 mg/mL of NO-np. Control cultures were grown in medium inoculated with 50 µL PBS. All cultures were incubated at 37°C with 5% CO_2_ at 100% humidity for 24 h. Tissue inserts were removed, fixed in 10% formalin for 24 h, processed, and embedded in paraffin. Four micron vertical sections were fixed to glass slides, stained with hematoxylin and eosin (H&E) and examined by light microscopy.

### Lactate Dehydrogenase (LDH) Measurements

The release of LDH from the HSF or RHE into the medium was monitored as a measure of cell damage. LDH in medium from cultures containing uninfected and infected tissues was measured at 24 h by CytoTox-ONE™ kit (Promega, Madison, WI) according to the manufacturer's instructions. MR*SA* 6498 cells alone incubated under identical conditions were included as negative controls. The LDH released in the presence of bacteria was expressed relative to the untreated control tissue with the LDH activity of MR*SA* cells alone subtracted from the LDH of the tissue plus the bacteria.

### *In Vivo* Abscess Model and NO-np Treatment

To investigate the antimicrobial efficacy of NO-np for subcutaneous abscesses formed by MR*SA*, female Balb/c mice (6 to 8 weeks old; National Cancer Institute, MD) were anesthetized with 100 mg/kg ketamine and 10 mg/kg xylazine, the hair on their flanks was shaved, and the skin disinfected with iodine. Then, a suspension of 100 µL with 10^7^ *SA* 6498 in PBS was inoculated subcutaneously in each flank of the animals (two abscesses per mouse). The next day (day 2), a suspension with 5 mg/mL of NO-np or np dissolved in PBS was injected into or topically applied above the abscesses. Mice receiving topical therapy were also treated with a second topical application of NO-np or np on day 3. Untreated, infected and non-infected mice were used as additional controls. On day 4, the mice were euthanized and the abscesses were excised, homogenized, and cultured quantitatively by plating on tryptic soy agar.

### Histological Processing

At day 4 after MR*SA* abscess formation after infection and treatment as above, abscess tissues were excised from euthanized mice, fixed in 10% formalin for 24 h, processed, and embedded in paraffin. Four micron vertical sections were fixed to glass slides and subjected to H&E, Gram, Gomori's trichrome, or CD34 staining to observe the tissue morphology, bacteria, collagen deposition, or vascularization, respectively. Slides were examined by light microscopy with an Olympus AX70 (Melville, NY) microscope, and images were obtained (QImaging Retiga 1300 digital camera [Burnaby, British Columbia, Canada]) with QCapture Suite V2.46 software (QImaging).

### Abscess Area Analysis

Abscess area analysis was performed at day 4 after infection. Each abscess was measured using a caliper and the area was determined.

### Cytokine Determinations

Mice were euthanized at day 4 post-infection and two abscesses per mouse were individually homogenized in 2 mL PBS in the presence of protease inhibitors (Complete Mini; Boehringer Ingelheim Pharmaceuticals Inc., Ridgefield, Connecticut, USA). The homogenates were centrifuged at 6,000 *g* for 10 minutes to remove cell debris, and the supernatant was frozen at −80°C until tested. The supernatants were assayed for IL-1β, IL-4, IL-10, IL-12p70, MCP-1, TNF-α, TGF-β, and IFN-γ using ELISA (Becton Dickinson Biosciences Pharmingen, San Diego, California, USA; and eBiosciences, San Diego, California, USA). The detection limits of cytokine assays are 7.8 pg/mL for IL-4 and MCP-1, 15.6 pg/mL for IL-1β and TNF-α, 31.3 pg/mL for IL-10 and IFN-γ, 60 pg/mL for TGF-β, and 62.5 pg/mL for IL-12p70, as stated by the manufacturer.

### Statistical Analysis

All data were subjected to statistical analysis using GraphPad Prism 5.0 (GraphPad Software, La Jolla, CA). *P* values were calculated by analysis of variance and were adjusted by use of the Bonferroni correction. *P* values of <0.05 were considered significant.
